# Analytic methodology for demographic variation analyses for wave 1 of the global flourishing study

**DOI:** 10.1186/s44263-025-00140-2

**Published:** 2025-04-30

**Authors:** R. Noah Padgett, Matt Bradshaw, Ying Chen, Richard G. Cowden, Sung Joon Jang, Eric S. Kim, Koichiro Shiba, Byron R. Johnson, Tyler J. VanderWeele

**Affiliations:** 1 Department of Epidemiology, Harvard T.H. Chan School of Public Health, Boston, MA USA; 2 Human Flourishing Program, Harvard University, Cambridge, MA USA; 3 Institute for Studies of Religion, Baylor University, Waco, TX USA; 4 Department of Psychology, The University of British Columbia, Vancouver, Canada; 5 School of Public Health, Department of Epidemiology, Boston University, Boston, MA USA; 6 Institute for Studies of Religion, Department of Sociology, Baylor University, Waco, TX USA; 7 School of Public Policy, Pepperdine University, Malibu, CA USA; 8 Departments of Epidemiology and Biostatistics, Harvard T.H. Chan School of Public Health, Boston, MA USA

**Keywords:** Global Flourishing Study, Methodology, Demographics, Meta-analysis, Heterogeneity

## Abstract

In this article, we describe the statistical and design methodology of the demographic variation analyses used as part of a coordinated set of manuscripts for wave 1 of the Global Flourishing Study (GFS). Aspects covered include the following: evaluating demographic variation, accounting for the complex sampling design, missing data and imputation, and meta-analysis. We provide a brief illustrative example of the demographic variation analyses using a measure of purpose in life from the GFS survey and conclude by outlining some strengths and limitations of the analytic and statistical methodology employed.

## Background

The Global Flourishing Study (GFS) is a large, multinational panel study that aims to explore the distribution, determinants, and interrelations of various concepts related to human well-being with more than 200,000 people across a geographically and culturally diverse set of countries around the world [1–3]. Interest in flourishing has surged in recent years across various fields like psychology, economics, and public health [4–10]. However, many aspects of well-being remain underexplored, especially globally, as much of the well-being literature has been shaped by Western perspectives [7, 11]. As a multinational panel study, the GFS provides an avenue to explore well-being and flourishing from a multicultural perspective to fill this gap.

The purpose of this article is to describe the methodology applied to the set of construct-specific *demographic variation* analyses that were produced using wave 1 data from the GFS, most of which are planned for inclusion in manuscripts that are being considered for publication as a coordinated set of manuscripts using Wave 1 of the GFS. These demographic variation analyses apply a common preregistration template principally focused on exploring the distribution of scores on indicators of aspects of flourishing in each country and describing demographic differences in these indicators across groups and countries.

Exploring demographic variation in aspects of flourishing provides a baseline for describing how individual differences may be related to flourishing from different angles. Many of the constructs included in the GFS are seldom included in cross-cultural cohort studies, see survey development report [12], providing a novel opportunity to gain multinational insights into various aspects of wellbeing. All analyses are conducted separately by country, which not only preserves potential heterogeneity in the interpretation of survey items across countries but allows the results to be contextualized considering the sociocultural particularities within each country. Then, country-specific results are pooled using meta-analytic techniques to summarize the distribution of the construct indicator. The analyses described in this article provide a template for evaluating the distribution of flourishing globally by maintaining a consistent methodology allowing for the comparability of results across aspects of flourishing.

There are three core components of the current article. First, we begin by providing a high-level description of the data and measures used in the demographic variation analyses. Next, we discuss aspects of the methodology, namely evaluating demographic variation, accounting for the complex sampling design, missing data and imputation, and meta-analysis. Lastly, we use the sense of purpose in life outcome (“I understand my purpose in life.”; 0 = Strongly disagree, 10 = Strongly agree; cf. [13]) to provide an illustrative example of the analyses and results that will be presented in the construct-specific demographic variation manuscripts, see Kim et al. [14] for more details on the purpose in life outcome.

### Global Flourishing Study data

Currently available Wave 1 GFS data includes nationally representative samples of the adult population (18 years old and older) from 22 geographically and culturally diverse countries, including Argentina, Australia, Brazil, Egypt, Germany, Hong Kong (Special Administrative Region of China), India, Indonesia, Israel, Japan, Kenya, Mexico, Nigeria, the Philippines, Poland, South Africa, Spain, Sweden, Tanzania, Turkey, the United Kingdom, and the United States (Wave 1 data will also become available for mainland China once Wave 2 data are released in early 2025). These countries were selected to (a) maximize coverage of the world’s population; (b) ensure geographic, cultural, and religious diversity; and (c) prioritize feasibility and existing data collection infrastructure. Data collection was carried out by Gallup, a global analytics and advisory organization with decades of experience collecting global data on various aspects of human life. Most of the data for Wave 1 were collected in 2023, with some countries beginning data collection in 2022; exact dates of data collection vary by country [15]. The GFS is set to continue with four additional waves of annual panel data collection from 2024 to 2027. The precise sampling design that was used to collect Wave 1 data varied by country to ensure nationally representative samples for each country. Further details of the sampling design methodology are available elsewhere [15, 16].

Survey items included numerous aspects of well-being such as happiness and life satisfaction, physical and mental health, meaning and purpose, character and virtue, close social relationships, and financial and material stability [13] along with numerous other demographic, social, economic, political, religious, personality, childhood, community, and health variables. Development of the GFS survey occurred over eight distinct phases: (1) selection of core well-being and demographic questions; (2) solicitation of social, political, psychological, and demographic questions from domain experts worldwide; (3) revision of the initial survey draft based on feedback from scholars around the world representing various academic disciplines; (4) modification of question items following input from experts in multinational, multiregional, and multicultural survey research; (5) survey draft refinement based on compiled input from an open invitation to comment, posted publicly and sent to numerous listservs; (6) questionnaire optimization with support from Gallup survey design specialists; (7) adaptation of items from an interviewer-administered to a self-administered survey instrument using best practices for web survey design to minimize item non-response, illogical responses, and incomplete responses; and (8) confirmation by scholars in several participating countries that translations accurately captured the intended meaning of each question [3, 16].

The data are publicly available through the Center for Open Science (https://www.cos.io/gfs). During the translation process, Gallup adhered to TRAPD model (translation, review, adjudication, pretesting, and documentation) for cross-cultural survey research [17]. Additional information about methodology and survey development can be found in the GFS Questionnaire Development Report [2, 12] as well as the GFS Methodology [15], GFS Codebook (https://osf.io/cg76b), and GFS Translations documents [18].

## Measures

### Demographic variables

A total of 9 demographic variables were considered. Seven of those—age, gender, marital status, employment, religious service attendance, education, and immigration status—were assessed with the same categories across all countries. Religious affiliation was assessed in all countries, but the response options varied considerably across countries. Racial/ethnic identity was assessed in some but not all countries, and response options were unique to each country. Details about the demographic items and response options for each are reported in the GFS Questionnaire Development Report [3] and the GFS Codebook (https://osf.io/cg76b). For the purpose of the demographic variation analyses, recorded responses of “don’t know,” “refused,” “skipped,” and “prefer not to answer” were coded as missing.

For each manuscript in which demographic variation analyses are reported, summary statistics of the demographic variables (i.e., counts and proportions) are based on non-imputed “raw” data using complex survey-adjusted estimates. Summary statistics for the 7 demographic variables that were measured consistently across the countries are provided for the whole dataset in the main text. Online supplemental material will report summary statistics for this set of demographic characteristics by country, with the addition of country-level religious affiliation and racial/ethnic identity (when available).

### Criterion/outcome variables

A range of continuous, binary, Likert-type, and nominal response scales were used to assess the different constructs included in the GFS. Means were estimated for approximately continuous variables. All Likert-type and nominal measures were recoded into binary variables (based on cutoffs defined a priori in the preregistrations), with proportions estimated for all binary variables.

### Evaluating demographic variation

This section describes the analyses which were carried out within each country. The following section describes random effects meta-analyses used to summarize results across countries. Analyses were implemented across multiple software packages (R [19], Stata [20], SAS [21], and SPSS [22] to ensure consistency in results and ease of use by the larger core group [23]). Implementing the analyses in separate software also allows for a greater reach across fields because other scholars can utilize and replicate our analyses in their software of choice. Any deviations across software packages implementation are described below.

### Separating analyses by country

The core analyses of the GFS were conducted separately within each country. As described below, summary statistics were obtained by random effects meta-analysis rather than for example by use of a multi-level model. A key advantage of this approach is that it does not presume cross-cultural measurement equivalence of the measures, which is important because most constructs were assessed using a single item and cognitive testing during the survey development process suggested some variation in the interpretation of items across countries [24, 25]. It is thus preferable to treat the measures as closely related, but not identical, assessments of each construct across the countries. We chose to conduct analyses separately for each country, which not only preserves potential heterogeneity in the interpretation of survey items across countries but allows the results to be contextualized in light of the sociocultural particularities within each country. This approach also aligns with our decision to use a random effects meta-analysis to combine estimates for each demographic category, which implies that the means/proportions we combine are not necessarily samples from an identical superpopulation but the estimates form a distribution of means/proportions that we aim to summarize.

### Country means/proportions (and Gini coefficient)

Computing each country’s overall estimated mean/proportion of the outcome variable is based on the sampling weighted mean/proportion of observations. The presence of strata in the sampling design of some countries leads to the weighted mean/proportion also being averaged over strata. Variances and standard errors were computed using the Taylor series method [26–28]. For continuous outcomes, the Gini coefficient was also reported. The Gini coefficient ranges from 0 to 1 [29], where the lower bound reflects complete equality (i.e., all the persons receive the same value on the outcome) and the upper bound reflects complete inequality (i.e., one person has the highest possible score on the outcome and all others have 0). The estimator used for the Gini coefficient is based on the generalized linearization variance estimator [30].

### Subgroup means and proportions

The main text of the manuscripts in which demographic variations are reported includes a table presenting the means/proportions of the measure for each demographic group category based on meta-analysis described below. The country-specific results will be reported in the online supplemental material of each manuscript. Within each country, we computed the within-group means/proportions. In general, the point estimates were obtained using a weighted mean/proportion. Subgroup estimates of variance, or standard error, for the estimated mean/proportion are such that the specific estimator used varies across countries because the sampling design differs based on whether strata are absent or present [31]. Interval estimates for the mean of continuous outcomes (ranged 0–10) were based on a Wald-type confidence interval where items were treated as continuous which could rarely lead to intervals exceeding the bounds of the observed range of values; in such cases we have advised authors to truncate the limits to be within the range of observed values. Some slight differences are present in implementations across R, SAS, SPSS, and Stata software.

The subgroup analyses incorporated a test of whether the mean/proportion varied between subgroups of the demographic characteristic. These tests were based on Wald-type test [32, 33]. These tests tended to be highly powered, especially in countries with larger sample sizes, and the resulting *p* values tended to be near 0. These country-specific tests were pooled across countries to report a “global *p* value” corresponding with a test of whether the outcome mean/proportion varied among subgroups for each demographic characteristic (see “Global* p* values” subsection below). Complementary forest plots of the pairwise differences for specific comparisons of interest may also be provided in the online supplements.

### Accounting for the complex sampling design

Accounting for the complex sampling design was accomplished by utilizing the information provided by Gallup on the primary sampling unit (PSU) IDs, strata IDs, and sampling weights. The weighting variable and PSU/strata IDs were included in all country-specific analyses. A complexity arises when respondents are recruited face-to-face, because sometimes this results in groups (strata) with only one PSU. When a stratum has only a single case, this is known as a lonely PSU, and makes variance and standard error estimation more complex because traditional methods assume multiple PSUs within each stratum [27, 34]. We elected to use the “certainty” specification where single-PSU stratum do not contribute to the variance; this maintained relatively comparable results across statistical software depending on the level of missingness in the demographic characteristic and on the specific outcome. Complete details concerning the implementation of these methods to account for the complex sampling design of each country can be found in the open code [35]. The methods were generally the same across all software packages, with very minor exceptions. Although detailed discussion of the methods that were used to account for the complex sampling design is beyond the scope of this article, additional information about the issues that were encountered can be found in Padgett and colleagues [23]. We mostly relied on the default settings within each software package, which led to nearly identical results across software packages, with slight differences, principally in standard errors, mainly attributable to the imputation of missing data.

### Missing data and multiple imputation

All missing variables are imputed using multivariate imputation by chained equations [36, 37]. The imputation model incorporated the criterion/outcome variable, all demographic characteristics, including race/ethnicity and religious affiliation when available, and sampling weights. The sampling weights were included as a variable in the imputation models. Including the sampling weight in the multiple imputation procedure allowed study missingness to be related to the propensity of being included in the study. We elected not to include strata as a predictor in countries where strata were available to avoid a singularity in the design matrix due to single-PSU strata. To account for variations in the assessment of certain variables across countries (e.g., race/ethnicity and religious affiliation), we conducted the imputation process separately for each country. The within-country imputation approach ensured that the imputation model accurately reflects country-specific contexts and assessment methods.

While conducting multiple imputation with five imputed datasets is a commonly used default [38], a more robust recommended number of imputations relates to the fraction of missing information (FMI) of the observed dataset [39]. The rate of missing data for this first wave of the GFS was quite low (< 5% for nearly all variables that were measured), and for the demographic characteristics in particular, the item with the largest percent missing was racial/ethnic identity at 1.6% (which is not being meta-analyzed due to varying response categories across countries). Across all the items used as demographic factors, the percent of respondents with any missing data was 3.2% (a rough approximation of the FMI is therefore 0.032). Using an efficiency argument (FMI/m ≤ 0.05) commonly used, the number of imputed datasets needed would be less than 3. In preliminary testing, we evaluated using more imputed datasets (*m* = 20) and found no meaningful differences in results compared to only 5 imputations or compared across software implementations of multiple imputation. Increasing past 5 imputed datasets was therefore thought to result in insufficient gains to justify the considerable increase in computational time due to the imputation being conducted separately by country and research team. However, we anticipate higher levels of missing data in subsequent waves due to wave-specific non-response, and analyzes should consider using at least 20 imputations in subsequent waves of data analysis in spite of the additional computing time.

### Meta-analysis

The 22 countries were chosen to have broad geographical, cultural, and religious coverage; the countries include all six populated continents and represent about half of the world’s population. The random effects meta-analysis would be interpreted as estimating the pooled mean/proportion and the standard deviation of the indicator means/prevalences from a hypothetical underlying population of which the sample of 22 countries would be representative. While such an underlying population is hypothetical, given the broad diverse coverage of the 22 countries, this was viewed as a reasonable target of interest. However, the results for each of the 22 countries are also provided, which are of interest in their own right, and may also be useful for readers who would prefer not to consider this underlying hypothetical population. Moreover, we provide a population weighted fixed effects meta-analysis to evaluate similar indicator prevalences/means where the principal target of inference concerns individual people in the 22 countries rather than the countries themselves.

### Preparing for meta-analyses

All meta-analyses were conducted in **R** [19] using the *metafor* package [40] through an open-source application developed for these analyses [35]. Please see https://wviechtb.github.io/metafor/ for more information on the *metafor* package. The effect size, or values to be meta-analyzed, depends on the scale of the outcome for a particular study. For continuous outcomes, the means were directly meta-analyzed using the within-country squared standard error as the variance estimate. For binary outcomes, the proportion was first converted using the logit transformation. A condition of this conversion is that the estimated within-country proportion must strictly be within the interval (0,1). To avoid the boundary constraint, we set bounds on the how small or large the estimated proportion could be for the purpose of meta-analysis based on the country sample sizes, that is $\widehat{p}\in \left(\frac{1}{N}, 1-\frac{1}{N}\right)$, where $N$ is the sample size of the country where $\widehat{p}$ was estimated. Estimated proportions outside these bounds replaced with the closest bound. This bound was very rarely needed because most proportions were between 0.2 and 0.8, but the bound was necessary in rare groups, such as the “other” gender group, for some (but not all) outcomes. The standard error of the logit transformed proportions was obtained using the delta-method:

$$SE\left(logit\left(\widehat{p}\right)\right)={\left(\frac{1}{N\times \widehat{p} \times (1-\widehat{p})}\right)}^{0.5},$$

This equation was used to represent uncertainty on the transformed scale for input into the meta-analyses. For countries with large sample sizes, the resulting logit standard error can be quite small ([41], p. 40, Eq. 3.5). Additionally, it is possible to meta-analyze the proportions directly pooling the estimates in the same manner as with the means for continuous outcomes. This alternative approach would down-weight the influence of more extreme proportions when pooling across countries compared to the logit transformation approach.

### Random effects meta-analysis

For all the core demographic GFS studies, a general random effects model was used. This model assumes that the effect sizes in the population follow a normal distribution [42–44], that is:

$${y}_{i}\sim \text{Normal}({y}_{i}^{*},{v}_{i})$$

$${y}_{i}^{*}\sim \text{Normal}(\theta ,{\tau }^{2})$$

where ${y}_{i}$ is the mean within each country, ${v}_{i}$ is the variance/uncertainty of ${y}_{i}$ within each country, ${y}_{i}^{*}$ is the unknown true mean for the subgroup within country, $\theta$ is the population mean for the subgroup, and ${\tau }^{2}$ is the estimated variance/heterogeneity of ${y}_{i}^{*}$. The model was estimated using the Paule and Mandel estimator [45–47].

Heterogeneity in the pooled means/proportions was assessed using the estimated standard deviation of the distribution of means/proportions ($\tau$). All substantive manuscripts will include a forest plot for all meta-analytic estimates, which provide a better indication of the heterogeneity, along with the Q-statistics and Q-profile confidence intervals for $\tau$. Additionally, prediction intervals for the country means/proportions described next provide a sense of the heterogeneity from the perspective of sampling variability at the country level.

### Prediction intervals

For each manuscript in which demographic variation analyses are reported, we included prediction intervals based on the calibrated effect size from the random effects meta-analyses. The calibrated effect size is computed based on the meta-analysis results following well-established methods [48–50] that use the following formula,

$${\widetilde{y}}_{i}=\widehat{\theta }+\left({y}_{i}-\widehat{\theta }\right){\left(\frac{{\widehat{\tau }}^{2}}{{\widehat{\tau }}^{2}+{v}_{i}}\right)}^{0.5},$$

where ${\widetilde{y}}_{i}$ is the calibrated effect size of country $i$. The calibrated effect sizes create prediction intervals [50]. A 95% prediction interval is an interval constructed so that the true mean/proportion for a randomly chosen country from the random effects distribution will fall within this interval 95% of the time. For binary outcomes, the prediction interval bounds were back-transformed from the logit scale to the proportion scale for reporting. Currently available Wave 1 GFS data includes a relatively low number of “studies” for a meta-analysis (i.e., 22 countries). Thus, we approximated the prediction interval bounds using the smallest and largest calibrated effect sizes resulting in a $\frac{k-1}{k+1}\times 100\%$ prediction interval in line with Wang and Lee’s method of approximating prediction intervals without extrapolating beyond the observed data. The bounds for the prediction interval are at an $\frac{<span class='convertEndash'><span class='convertEndash'><span class='convertEndash'>22-1</span></span></span>}{22+1}\times 100\approx$ 91% confidence level for most outcomes. We say *most* outcomes because some subgroup means/proportions were not estimable in some countries with smaller sample sizes. For example, in Egypt, we could not estimate the mean/proportion for the “other” gender group because no participants were in this category.

### Population weighted meta-analysis

A fixed effects meta-analysis was conducted as a supplemental analysis to the random effects meta-analysis described above, providing an opportunity for researchers to consider both sets of results depending on which interpretative approach is most appropriate for their purposes. Inferences focused on differences across countries may utilize the random effects estimates, as these align with the target of inference, whereas analyses giving individuals equal weight align more with the results of the supplemental fixed effects meta-analyses. While the random effects meta-analysis assumes a distribution over the subgroup means/proportions across countries relaxing the measurement invariance assumption somewhat, the supplemental fixed effects does not a assume a distribution over the values meta-analyzed but more directly estimates the weighted average over countries where the weight in this analysis is the total 2023 population (rather than the observed sample size) within each country. Note the fixed effects approach taken here essentially estimates the effect across individuals in the various countries, and can be given this interpretation even if there is heterogeneity across countries in effect sizes [51]. The meta-analytic estimate is

$$\widehat{\theta }=\frac{\sum {w}_{i}{y}_{i}}{\sum {w}_{i}},$$

where ${y}_{i}$ is mean/proportion of the outcome within a subgroup for each country and ${w}_{i}$ is the weight for each country. A common choice for the weight is the inverse of the sampling variance ${v}_{i}$, but in this analysis we aimed to estimate the overall average by treating individuals with equal weight instead of countries with equal weight, and without assuming a common mean across countries. We therefore once again used a weight for each country that scales based on the total 2023 population size of each country.

Using the population sizes provided by Gallup, the fixed effects meta-analysis estimated the average subgroup mean/proportion, weighted by the population size of each country. The country sizes used to create weights are shown in Table 1.

**Table 1:** Gallup provided estimates of population sizes for population weighted meta-analysis

Country	Population Est	Est. % of age 18 +	Est. population size of age 18 +	Meta-analysis weight
Argentina	35,576,161	0.93	33,085,830	0.014
Australia	21,255,952	0.96	20,329,009	0.008
Brazil	171,666,633	0.91	156,216,636	0.064
Egypt	74,517,704	0.75	56,059,669	0.023
Germany	72,343,802	0.96	69,392,175	0.028
Hong Kong (S.A.R. of China)	6,461,584	0.94	6,097,316	0.003
India	1,058,538,924	0.91	964,761,394	0.396
Indonesia	206,057,132	0.94	193,828,725	0.080
Israel	6,869,826	0.90	6,191,774	0.003
Japan	110,582,052	0.97	107,139,250	0.044
Kenya	33,598,920	0.76	25,639,336	0.011
Mexico	96,256,568	0.93	89,518,608	0.037
Nigeria	124,471,001	0.88	109,534,481	0.045
Philippines	80,502,849	0.91	73,216,028	0.030
Poland	31,870,824	0.97	30,917,886	0.013
South Africa	42,793,704	0.91	38,728,302	0.016
Spain	41,045,172	0.95	39,144,781	0.016
Sweden	8,641,262	0.96	8,296,398	0.003
Tanzania	37,099,584	0.85	31,482,707	0.013
Turkey	65,514,213	0.96	62,703,653	0.026
United Kingdom	55,273,701	0.96	53,129,081	0.022
United States	273,430,984	0.95	259,759,435	0.107

*S.A.R.* Special Administrative Region

### Global p values (combining p values from country-specific tests)

The harmonic mean *p* value was used to combine *p* values across different countries [52, 53]. The combined *p* value was used to test the null hypothesis of no differences in the mean/proportion for each construct indicator among subgroups in all countries, against the alternative hypothesis that in at least one country the mean/proportion differs among subgroups defined by that demographic variable. The harmonic mean *p* value method is more robust to dependency among pooled *p* values [53]. Although the country-specific tests are technically independent—an underlying assumption of most classic approaches to pooling *p* values [54]—assuming independence of the *p* values may not be entirely tenable given a common underlying set of items, translation procedures, data cleaning techniques, and imputation models. To account for multiple testing, we present Bonferroni-corrected *p* value thresholds for the meta-analytic results based on the number of demographic variables included [55, 56] in the primary meta-analytic results (corrected threshold $\alpha =0.007$). The Bonferroni adjustment for multiplicity was applied to the significance level cutoff (alpha) and not the *p* values (we divided alpha by the number of tests and not multiplying the *p* values by the number of tests). Providing the standard 0.05 significance threshold and Bonferroni-adjusted significance threshold provides transparency in how multiplicity was considered. However, the reported harmonic mean *p* value is relatively robust to multiple testing already maintaining a constant Type-I error rate regardless of the number of tests being conducted [53].

### Example analysis—purpose in life

We will illustrate the aforementioned methodology and analyses and corresponding results with an example concerning understanding one’s purpose in life; see Kim et al. [14] for further details.

### Construct overview and importance

A sense of purpose in life, the extent that people see their lives as having a sense of direction and goals that are anchored in core values, is a central component of human well-being [57, 58]. This factor is important in its own right, but it is also important because it shapes people’s trajectories of psychological, social, behavioral, spiritual, and physical health [59–67]. One indicator of purpose in life in the GFS survey is the item, “I understand my purpose in life,” self-reported from 0 = Strongly disagree to 10 = Strongly agree [13], which will be used to illustrate the analytic approach.

### Illustrative results

Table 2 displays the nationally representative descriptive statistics for the 7 demographic variables of the entire observed sample that were assessed consistently across the 22 countries included in Wave 1 of the GFS (*N* = 202,898). Participant ages ranged the entire adult lifespan (18–80 +). The gender distribution was nearly balanced across female (51%) and male (49%), along with a small representation from other gender identities (0.3%). Most participants were married (53%), attained 9–15 years of education (57%), native-born (94%), and employed for an employer (39%). Regular attendance at religious services varied, with most never attending (37%), some attending once a week (19%), and others attending once a week or more (13%).

**Table 2:** Demographic characteristics of the Global Flourishing Study sample (wave 1)

**Characteristic**	***N*** = **202,898**^a^
**Age group**	
18–24	27,007 (13%)
25–29	20,700 (10%)
30–39	40,256 (20%)
40–49	34,464 (17%)
50–59	31,793 (16%)
60–69	27,763 (14%)
70–79	16,776 (8.3%)
80 or older	4,119 (2.0%)
(Missing)	20 (< 0.1%)
**Gender**	
Male	98,411 (49%)
Female	103,488 (51%)
Other	602 (0.3%)
(Missing)	397 (0.2%)
**Marital status**	
Married	107,354 (53%)
Separated	5195 (2.6%)
Divorced	11,654 (5.7%)
Widowed	9823 (4.8%)
Single, never married	52,115 (26%)
Domestic Partner	14,931 (7.4%)
(Missing)	1826 (0.9%)
**Employment status**	
Employed for an employer	78,815 (39%)
Self-employed	36,362 (18%)
Retired	29,303 (14%)
Student	10,726 (5.3%)
Homemaker	21,677 (11%)
Unemployed and looking for a job	16,790 (8.3%)
None of these/Other	8431 (4.2%)
(Missing)	793 (0.4%)
**Religious service attendance**	
More than 1/week	26,537 (13%)
1/week	39,157 (19%)
1–3/month	19,749 (9.7%)
A few times a year	41,436 (20%)
Never	75,297 (37%)
(Missing)	722 (0.4%)
**Education**	
Up to 8 years	45,078 (22%)
9–15 years	115,097 (57%)
16 + years	42,578 (21%)
(Missing)	146 (< 0.1%)
**Immigration status**	
Born in this country	190,998 (94%)
Born in another country	9791 (4.8%)
(Missing)	2110 (1.0%)
**Country**	
Argentina	6724 (3.3%)
Australia	3844 (1.9%)
Brazil	13,204 (6.5%)
Egypt	4729 (2.3%)
Germany	9506 (4.7%)
Hong Kong (S.A.R. of China)	3012 (1.5%)
India	12,765 (6.3%)
Indonesia	6992 (3.4%)
Israel	3669 (1.8%)
Japan	20,543 (10%)
Kenya	11,389 (5.6%)
Mexico	5776 (2.8%)
Nigeria	6827 (3.4%)
Philippines	5292 (2.6%)
Poland	10,389 (5.1%)
South Africa	2651 (1.3%)
Spain	6290 (3.1%)
Sweden	15,068 (7.4%)
Tanzania	9075 (4.5%)
Turkey	1473 (0.7%)
United Kingdom	5368 (2.6%)
United States	38,312 (19%)

*S.A.R.* Special Administrative Region

^a^Weighted statistics computed using the tbl_svysummary function of the gtsummary package in R [19]

Table 3 provides the overall country-specific estimates of purpose ordered by magnitude, along with the 95% confidence interval of the mean, standard deviation, and Gini coefficient. A similar table is presented in all construct-specific manuscripts that include demographic variation analyses following the template reported in this article. Caution should be applied when comparing means/proportions across countries because of differences in translation across languages, cultural differences in response styles, and potential seasonal variation; assessments were made in different countries during different times of the year, and this variation in timing might also influence results.

**Table 3:** Ordered mean of purpose in life score of each country

Country	Mean	95% CI	SD	Gini
1. Indonesia	8.87	(8.81, 8.94)	1.67	0.09
2. Kenya	8.51	(8.43, 8.59)	2.51	0.13
3. Philippines	8.45	(8.38, 8.51)	2.02	0.12
4. Mexico	8.44	(8.37, 8.51)	1.96	0.11
5. Israel	8.44	(8.30, 8.58)	1.82	0.11
6. Nigeria	8.36	(8.28, 8.44)	2.11	0.13
7. South Africa	8.07	(7.94, 8.19)	2.40	0.15
8. India	8.04	(7.97, 8.12)	2.99	0.18
9. Tanzania	8.02	(7.90, 8.14)	2.96	0.18
10. Egypt	7.96	(7.87, 8.05)	2.41	0.16
11. Argentina	7.92	(7.84, 8.00)	2.32	0.15
12. Brazil	7.80	(7.75, 7.86)	2.50	0.17
13. Poland	7.77	(7.64, 7.91)	1.99	0.14
14. Spain	7.34	(7.27, 7.41)	2.26	0.17
15. Hong Kong (S.A.R of China)	7.24	(7.14, 7.35)	2.14	0.16
16. Turkey	7.20	(7.01, 7.39)	2.97	0.22
17. Germany	7.08	(7.02, 7.14)	2.34	0.18
18. United States	6.99	(6.92, 7.06)	2.59	0.20
19. Australia	6.85	(6.74, 6.96)	2.53	0.20
20. Sweden	6.65	(6.60, 6.70)	2.54	0.21
21. United Kingdom	6.64	(6.54, 6.75)	2.64	0.22
22. Japan	5.67	(5.64, 5.71)	2.29	0.22

*N*= 202,898

*CI* confidence interval, *SD* standard deviation, Gini index of inequality, *S.A.R.* Special Administrative Region

Countries from Asia (e.g., #1 Indonesia, #3 Philippines), Africa (e.g., #2 Kenya, #6 Nigeria, #7 South Africa, #9 Tanzania), and Latin America (e.g., #4 Mexico) dominated the top 10 rankings. This diversity suggests that high levels of understanding one’s purpose in life can transcend geographical and cultural boundaries, indicating an ability to achieve purpose in diverse circumstances. Traditionally high-income and more individualistic countries like #14 Spain, #16 Turkey, #17 Germany, #18 the United States, #19 Australia, #20 Sweden, and #21 the United Kingdom were lower in the rankings. This trend suggests that societies with strong community bonds, familial connections, rich cultural heritage, and perhaps a greater reliance on spiritual or religious frameworks tend to report higher levels of understanding one’s purpose in life. See Kim and colleagues [14] for further results and interpretation, along with an analogous set of results concerning another item on meaning in life.

**Fig. 1:**
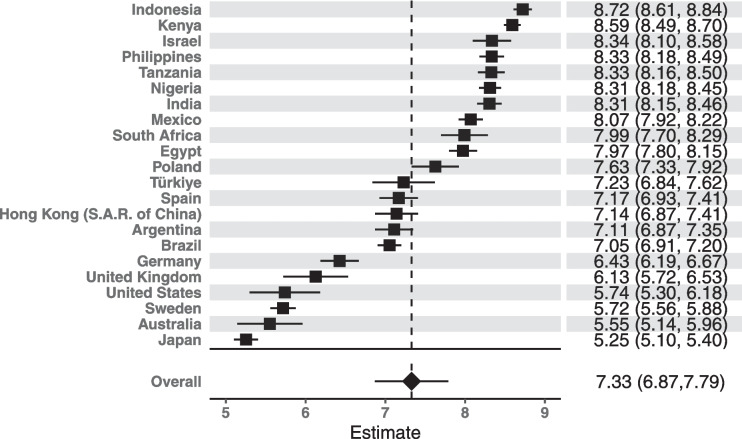
Forest plot for the meta-analysis of purpose in life mean scores for 18–24 years age category

Next, the results of the random effects meta-analyses that combined the subgroup means for the 7 demographic variables that were assessed consistently across all 22 countries are presented in Table 4. Religious affiliation and racial/ethnic identity were not measured consistently across the countries, so these demographic characteristics were not included in the meta-analyses. Each row of Table 4 represents a unique meta-analysis, totaling 34 in all; the online supplemental material in each manuscript will have a corresponding forest plot for each demographic category included in the meta-analysis, which displays the meta-analyzed effect estimate and the country-specific effect estimates. An example forest plot for the meta-analysis of mean purpose in life scores across countries for the 18–24 years age category is shown in Fig. 1.

**Table 4:** Random effects meta-analyses for purpose in life outcome means by demographic category

Variable category	Mean	95% CI	SE	Prediction Interval				Global
				LL	UL	Heterogeneity(τ)	$I^{\mathit2}$	*p* value
Age group								< .001**
18–24	7.33	(6.87, 7.79)	0.23	5.26	8.72	1.08	99.3	
25–29	7.41	(6.98, 7.84)	0.22	5.27	8.92	1.02	99.0	
30–39	7.54	(7.12, 7.96)	0.21	5.20	9.03	0.99	99.5	
40–49	7.62	(7.25, 8.00)	0.19	5.21	8.98	0.89	99.2	
50–59	7.70	(7.37, 8.04)	0.17	5.41	8.78	0.79	98.9	
60–69	7.79	(7.48, 8.09)	0.16	5.94	8.75	0.72	98.6	
70–79	7.82	(7.56, 8.08)	0.13	6.58	8.90	0.58	96.8	
80 or older^a^	7.88	(7.59, 8.16)	0.15	6.52	9.06	0.61	90.6	
Gender								< .001**
Male	7.66	(7.33, 7.99)	0.17	5.58	8.92	0.79	99.6	
Female	7.65	(7.32, 7.98)	0.17	5.77	8.83	0.79	99.6	
Other^a^	5.90	(5.07, 6.74)	0.43	3.94	7.76	1.40	86.9	
Marital status								< .001**
Married	7.89	(7.61, 8.17)	0.14	5.98	8.93	0.67	99.5	
Separated	7.47	(7.10, 7.84)	0.19	5.89	8.80	0.81	94.3	
Divorced	7.53	(7.17, 7.89)	0.18	5.54	9.05	0.82	96.9	
Widowed	7.81	(7.52, 8.10)	0.15	6.54	8.73	0.66	94.9	
Domestic partner	7.54	(7.03, 8.05)	0.26	5.17	9.63	1.15	99.2	
Single, never married	7.27	(6.78,7.75)	0.25	4.80	8.66	1.15	99.6	
Employment status								< .001**
Employed for an employer	7.67	(7.31,8.03)	0.18	5.49	8.85	0.86	99.6	
Self-employed	7.84	(7.57,8.12)	0.14	6.02	9.02	0.65	98.4	
Retired	7.80	(7.54,8.06)	0.13	6.22	8.61	0.60	97.9	
Student	7.32	(6.86,7.78)	0.24	5.42	8.88	1.09	98.4	
Homemaker	7.68	(7.37,7.99)	0.16	6.00	8.87	0.72	98.1	
Unemployed looking for a job	7.08	(6.54,7.62)	0.28	4.23	8.73	1.28	99.0	
None of these/other	7.24	(6.76,7.72)	0.24	4.91	8.70	1.10	97.3	
Education								< .001**
Up to 8 years	7.65	(7.28,8.02)	0.19	5.14	8.94	0.87	98.9	
9–15 years	7.67	(7.30,8.04)	0.19	5.57	8.82	0.88	99.7	
16 + years	7.83	(7.49,8.17)	0.17	6.04	9.36	0.81	99.4	
Religious service attendance								< .001**
> 1/week	8.49	(8.28,8.71)	0.11	7.34	9.24	0.50	97.1	
1/week	8.10	(7.93,8.28)	0.09	7.24	8.83	0.41	96.5	
1–3/month	7.80	(7.57,8.03)	0.12	6.32	8.79	0.54	96.3	
A few times a year	7.61	(7.32,7.90)	0.15	5.95	8.70	0.69	98.8	
Never	7.19	(6.85,7.53)	0.17	5.52	8.71	0.80	99.4	
Immigration status								< .001**
Born in this country	7.65	(7.31,7.98)	0.17	5.67	8.87	0.80	99.8	
Born in another country	7.55	(7.24,7.86)	0.16	6.03	9.18	0.68	94.1	

*N* = 202,898. *p < .05; **p < .007 (Bonferroni corrected threshold)

^1^SE Analogue standard error approximated by CI Width/4, CI confidence interval, LL lower limits of prediction interval, UL upper limit of prediction interval; prediction interval is the range of likely values of the estimate for a randomly selected country; τ is the standard deviation of the distribution of proportions across countries, which is an indicator of cross-national heterogeneity; I^2^ is an estimate of the variability in means due to heterogeneity across countries vs. sampling variability which is not uncommonly nearly 100% when there is nice precision in estimated proportion within country; and the Global p value corresponds to a test of the null hypothesis that there are no differences between the groups for that sociodemographic characteristic in all of the 22 countries

^a^Group is very small (< 0.1% of the observed sample) within several countries leading to large uncertainty in this estimate—be cautious about interpreting this estimate

Additionally, the forest plots are constructed such that all means/proportions are ordered by magnitude, and the y-axis varies to allow for a quick inspection of which countries have a high or low relative mean/proportion and whether these orders are similar across demographic categories. In addition to the forest plots for each mean/proportion, the online supplemental material of each manuscript will report the summary statistics (weighed counts/proportions) of each demographic characteristic and the weighted subgroup means/proportions for each country separately. These alternative groupings of results by country allows for comparison across demographic groups within a country, whereas the forest plots allow for comparison within demographic groups across countries. The meta-analyses using a population weighted (fixed effects) approach to estimate effects if we were to weigh within-country results by the size of the population the sample represents. Those results are not shown here but are reported in the online supplemental material of each individual manuscript.

### Strengths and limitations

The analytic methodology employed for the study of demographic variation of flourishing has several strengths and limitations that should be noted. A notable strength is the broad population coverage of the GFS. The countries included in Wave 1 of the GFS encompass approximately 64% of the world’s population [3]. Most of the analytic methods employed are relatively well-established, with a long history of being used in either epidemiology, public health, psychology, or sociology. We aimed to employ rigorous methods that appropriately incorporate the unique complex sampling design used in each country to obtain robust standard errors. All the code to reproduce analyses is openly available in several languages (R, SAS, SPSS, and Stata) for researchers to explore these data and results. All analyses were conducted at the country-level before being pooled using meta-analytic techniques to account for uncertainty in the estimates and quantify heterogeneity across countries. We used a random effects “distribution of effects” perspective of meta-analytic methods for our primary analyses but also reported a fixed effects “population weighted” perspective as a supplemental analysis. Using different theoretical perspectives for pooling estimates provides flexibility to the reader to interpret which set of effects are appropriate for their purposes.

There are limitations to consider as well. Sources of heterogeneity in the weighted subgroup means across countries could be due to seasonality effects, differences in interpretation, differences due to quality of translation, differences in mode of data collection, differences in the process and variables used for constructing respondent level weights, and other possible reasons depending on the specific construct of interest [16]. Most of the psychosocial constructs that were assessed only had a single item to represent the overall construct (e.g., sense of purpose in life), many of which were assessed with binary or ordinal response scales with few categories. However, it is not uncommon for such items to be used in large-scale epidemiologic studies such as the GFS, and decisions about which items and response scales to use were guided by several carefully planned phases of survey development [12]. These limitations result in some measures in the GFS survey to not be a suitable fit for answering certain research questions. The use of single-item assessments provides less construct coverage and generally lower true-score reliability, resulting in less power to detect differences across demographic categories [68]. The obtained country sample sizes for Wave 1 were relatively large (ranging from ~ 1500 to 38,000), which helps to mitigate this concern to a degree.

Several limitations on the side of the statistical methods employed are noted next. The precise implementations of the methods to account for the complex sampling design can sometimes be not fully transparent, especially in software packages that require a license (e.g., SAS, Stata). The use of several software packages helped to identify the effects of any software-specific peculiarities. A common issue we needed to deal with involved handling “lonely PSUs” [34], but we aimed to always use a “certainty” specification that fixed the variance contribution to zero in such cases when estimating variance components. This approach has the limitation of potentially underestimating the variance, or standard error, for a particular estimate. However, to the best of our knowledge, there is no generally agreed upon approach for handling such instances, and our aim is to be transparent about these decisions to reduce non-reproducibility because of unclear analytic decisions and researcher degrees of freedom [69].

The analyses outlined in this article are relatively straightforward, but also varied to allow for multiple interpretive lenses to be applied (e.g., within-country vs. cross-country patterns). Implementing these coordinated analyses has its challenges, such as complications implementing analyses using complex sampling weights, multiple imputation, and meta-analysis across several statistical packages, and yet we found remarkably similar results across packages in spite of slightly different implementations [23].

## Conclusions

The current article provides a description of the methods used in manuscripts reporting demographic variation analyses that leverage Wave 1 of the GFS, most of which are being considered for publication as a coordinated set of manuscripts based on the GFS. Using nationally representative data from 22 geographically and culturally diverse countries around the world, the set of planned demographic variation analyses of construct indictor prevalences/means related to well-being can play a role in identifying potentially vulnerable populations with lower well-being and also in identifying trends across countries and eventually also over time. The trends identified in the demographic variation analyses, supported by the methods described in this article, may also help shape the development of future interventions or policies aiming to promote well-being specifically for those vulnerable populations. However, the demographic differences themselves are purely descriptive and should not be interpreted causally. The interested reader is referred to our companion article, *Analytic Methodology for Childhood Predictors Analyses for Wave 1 of the Global Flourishing Study* [70], for a description of the methods used in the childhood predictors analyses of GFS outcomes manuscripts in the special collection.

## Data Availability

Data for Wave 1 of the GFS is available through the Center for Open Science (https://www.cos.io/gfs) upon submission of a pre-registration, and will be openly available without pre-registration beginning February 2025. Subsequent waves of the GFS will similarly be made available. Please see https://www.cos.io/gfs-access-data for more information about data access. Code for the GFS demographic variation analyses in multiple software is openly available (10.17605/osf.io/vbype).
